# Molecular Photosynthesis Research Facilitating Technology Development Towards Enhanced Indoor Farming

**DOI:** 10.1111/ppl.70407

**Published:** 2025-07-12

**Authors:** Pauli Kallio, Grzegorz Konert, Samuli Pyytövaara, Mikko Tikkanen

**Affiliations:** ^1^ Department of Life Technologies, Molecular Plant Biology University of Turku Turku Finland

## Abstract

Plants harness light energy through photosynthesis, a biological process that converts electromagnetic radiation into chemical form and drives CO_2_ fixation to produce biomass. Photosynthetic machinery, the engine of the process, is a complex network of protein assemblies that function in plant chloroplasts and control the energy conversion process under constantly changing environmental conditions. This machinery is responsible for practically all food production on Earth, yet the molecular details and constraints that affect the overall energy efficiency are often ignored in the context of farming applications. This review is targeted at a wide audience and provides insight into the basic mechanistic concepts of photosynthesis and how these connect plant growth, conditional acclimation and efficiency. We aim to explain how different lights affect the photosynthetic performance and interlink with other environmental variables, and discuss why this should be taken into account under artificial conditions. We believe that a science‐based view of future development that takes advantage of the molecular level knowledge on photosynthesis can be used for improved research equipment design and in commercial indoor farming applications with LED light technology and automated condition control. This requires fluent interdisciplinary communication from engineers who design research instrumentation to software developers and modelling experts involved in biological data processing. To advance this collaboration, we hope that this review serves as a bridge for those who are entering the field of molecular photosynthesis research, or people who are not specialised in plant science, but use or develop indoor farming and LED technologies.

## 1 Need to Combine Molecular Photosynthetic Research and Development of Farming Technologies

Photosynthesis is a biological process in which plants, green algae and cyanobacteria convert solar radiation into chemical energy. These organisms use light energy to incorporate (i.e., fix) CO_2_ into a range of different organic carbon‐based compounds that are essential for life as we know it. Although the fundamental principles in photosynthesis are the same in all photoautotrophic organisms, there are distinct structural and functional differences in the organisation and regulation of the systems, especially between prokaryotes and eukaryotes (Nikkanen et al. 2021). Photosynthesis is the primary source of basically all the biomass on Earth, including our food. Global food production still relies on traditional outdoor farming, while indoor farming technologies that utilise artificial lights are becoming more important for securing increasing food demand under the ongoing climate change (Asseng et al. 2020). This article aims to provide a general overview of the molecular mechanisms that drive plant photosynthesis and a starting point for those who would like to have a more detailed scientific view of perhaps the most important single biological process on Earth. We will explore the intersection of plant research and indoor farming applications, highlighting the need for more precise control over environmental conditions.

Notably, the natural growth conditions outside are usually very different from those in indoor farming systems. This needs particular attention when new solutions are designed. The energy efficiency of indoor farming is currently far from optimal and could be significantly enhanced through technology development that considers the molecular properties of the photosynthetic machinery that is driving this process (van Delden et al. 2021). Modern crop improvement based on genome editing relies on the same molecular principles and utilises the biochemical expertise we have on the photosynthetic organisms (Abdelrahman et al. 2021). The grand challenge is that photosynthesis is an extremely complex biological process and is dependent on the combined effect of environmental conditions and genetic architecture that regulate and protect the system (Tikkanen and Aro 2014; Hippler et al. 2021). The photosynthetic efficiency is ultimately determined by external conditions, including light intensity and spectrum, availability of CO_2_ and water, nutrients, temperature, humidity, together with internal factors, such as genetic background and the developmental stage of the plant (Slattery and Ort 2021; Sembada et al. 2024). Optimising the process for maximal photosynthetic efficiency is therefore a complicated practical task that needs to look beyond the traditional approaches. We already have a reasonably good understanding of the function and regulation of photosynthetic organisms in laboratory conditions, but currently, there are technological limitations that hinder research‐based innovations. It is crucial to know how plants use light for driving photosynthesis and what the associated main challenges are in developing new technologies to boost plant research, plant breeding and the design of next‐generation indoor farming technologies.

## 2 How Does Photosynthesis Work?

**FIGURE 1:**
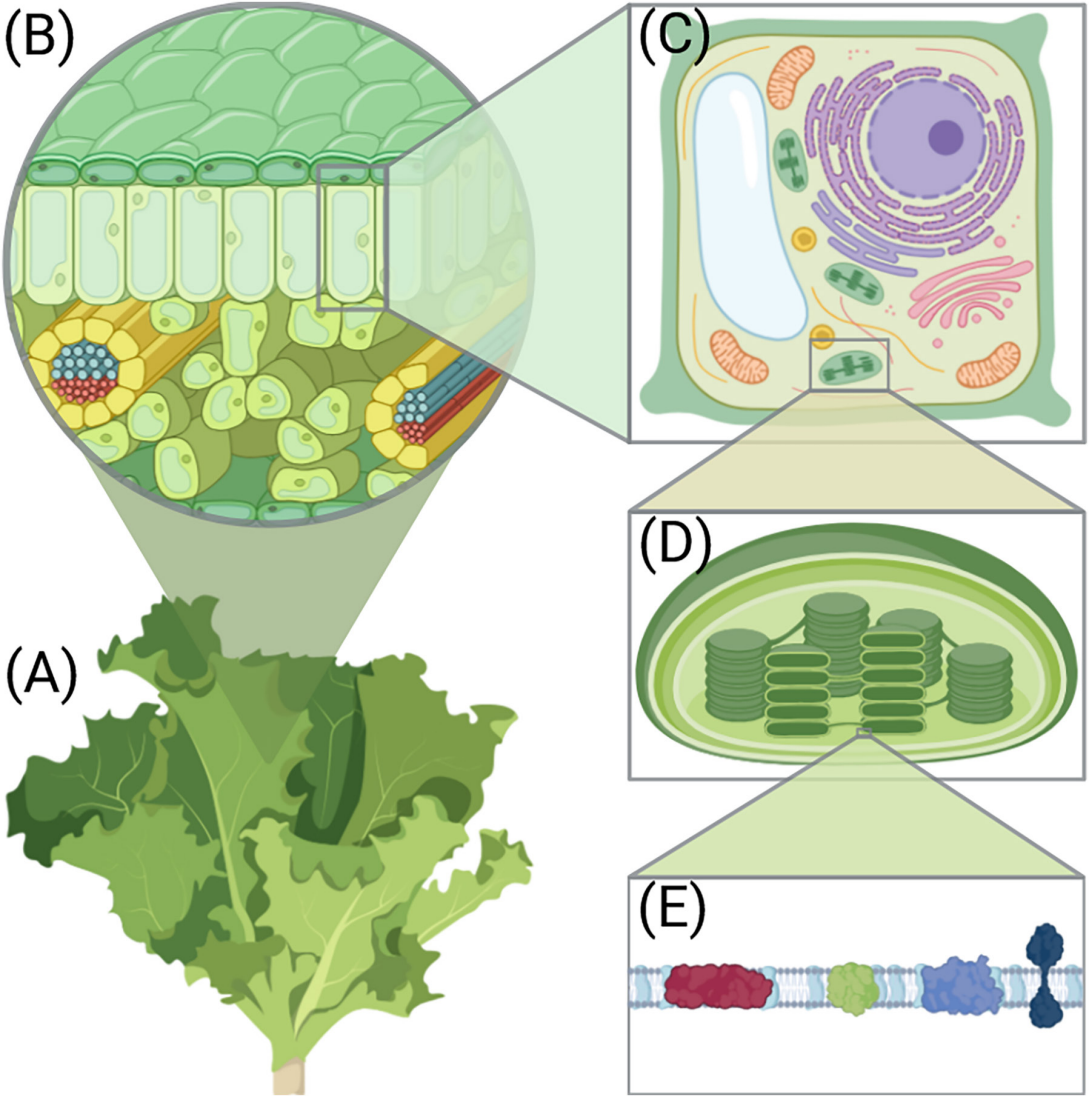
Schematic illustration of the plant structure and the location of the photosynthetic machinery in the leaf. (A) The visible green parts of a plant (here represented by *Lactuca sativa* ) are responsible for light harvesting and photosynthesis (B) cross section of a plant leaf showing its internal anatomy and different cell types. (C) Chloroplast‐containing mesophyll cell and its major organelles. (D) Cross section of a chloroplast showing the thylakoid membranes and their division into stacked grana membranes and connecting stoma lamellae. (E) Representation of the main components of the electron transport chain embedded in the thylakoid membrane.

The majority of the electromagnetic solar energy that reaches the Earth is in the near ultraviolet, visible and near infrared regions (150–4000 nm) of the spectrum. While the photosynthetically active radiation (PAR) covers approximately the 400–700 nm band of the solar radiation spectrum that is also visible for human eye (VIS), the concept of ‘optimal light spectrum’ for plant growth is dynamic and affected by multiple overlapping factors at the molecular level (Paradiso and Proietti 2022; Zhen et al. 2022). To understand this, we must look into the transfer and conversion of energy during the photosynthetic process (Foyer et al. 2012; Mirkovic et al. 2017; Rantala, Rantala, and Aro 2020). Photosynthesis takes place in the chloroplasts present in all the green cells of the plant (Figure 1). Chloroplasts enclose a membrane called the thylakoid membrane, where protein complexes are responsible for converting light energy into chemical energy (Ostermeier et al. 2024). The soluble nonmembrane part of the chloroplast contains the machinery that subsequently uses the generated chemical energy to fix carbon dioxide in order to produce sugars.

**FIGURE 2:**
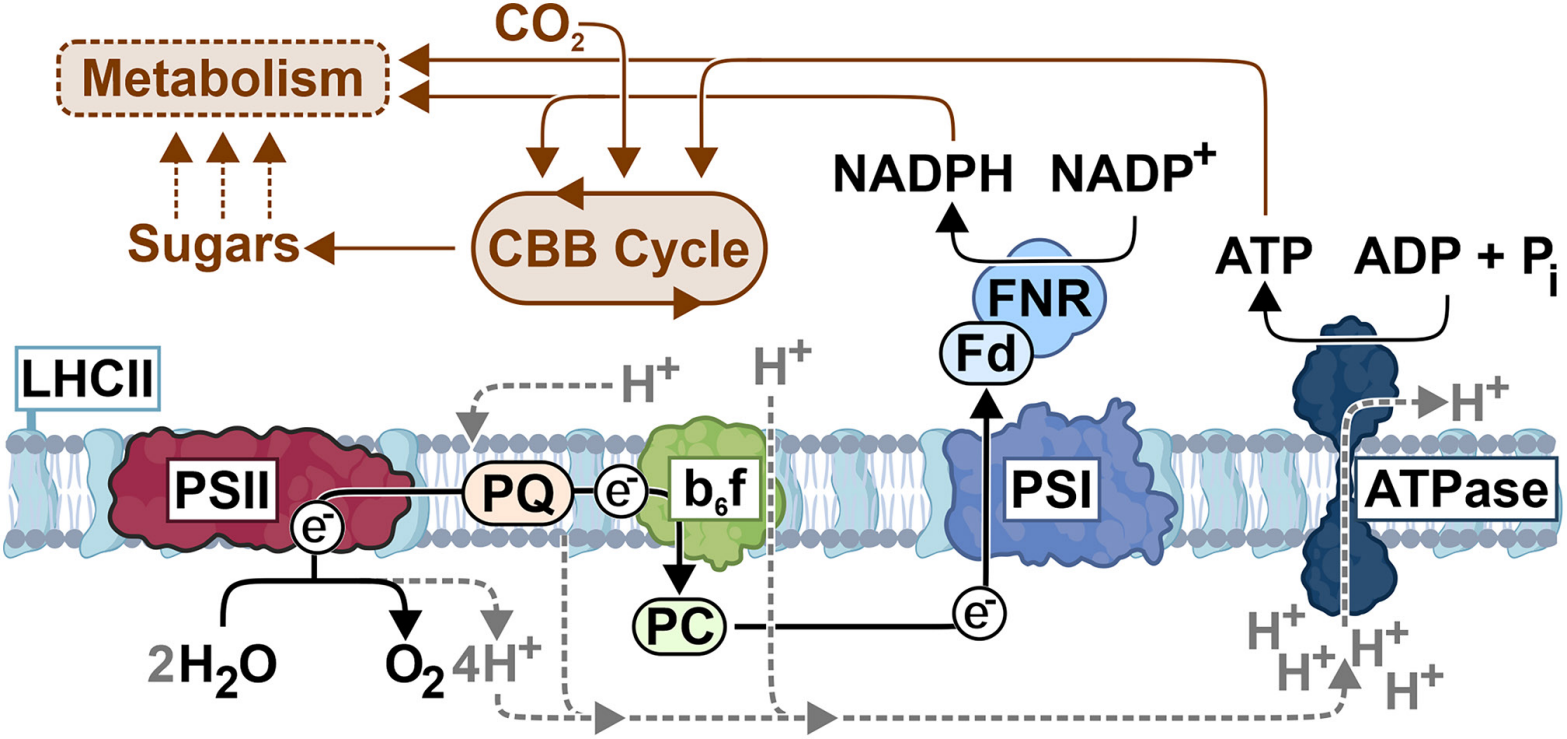
Schematic illustration of the major photosynthetic protein complexes necessary for the conversion of light energy into chemical energy and the use of this energy for CO_2_ fixation and chloroplast metabolism. ATPase, ATP synthase; b_6_f, cytochrome b_6_f; CBB, calvin benson bassham cycle; Fd, ferredoxin; FNR, ferredoxin NADPH reductase; LHCII, light harvesting complex II; PC, plastocyanin; PQ, plastoquinone; PSI, photosystem I; PSII, photosystem II.

The capture of light energy is primarily conveyed by chlorophyll molecules, green pigments that are excited by the incoming light. The efficiency of using the different spectral components of the light (i.e., the specific excitation wavelengths) depends on the structure and interactions of the thylakoid protein complexes that bind the chlorophylls (Maity and Kleinekathöfer 2023). In the thylakoid membrane, light energy is received by the light‐harvesting complexes (LHC) that transfer the energy from the light‐excited chlorophylls to photosystems II and I (PSII and PSI) (Figure 2). This machinery ultimately enables the conversion of the excitation energy into chemical form. At the core of the process, the harvested energy is funnelled to the PSII reaction center where it catalyses the breakdown of water (H_2_O) into protons (H^+^) and electrons (e^−^), releasing oxygen (O_2_) as a byproduct. The excited electrons that now carry the energy are transferred down the electron transfer chain (ETC) consisting of the plastoquinone (PQ) pool, cytochrome b_6_f protein complex (Cyt b_6_f) and plastocyanin (Pc) to PSI (Figure 2). In the conventional linear electron transport (LET) the water‐derived electrons are excited again at PSI and used for converting NADP^+^ (nicotinamide adenine dinucleotide phosphate) into NADPH, which supplies electrons and protons in many enzyme‐catalysed processes in the cell. In parallel, the protons released from the water splitting reaction are used to create a proton gradient (∆pH) across the thylakoid membrane. This gradient drives the enzymatic reactions of ATP synthase to produce ATP, the primary chemical energy carrier of all living cells. Together, NADPH and ATP power the photosynthetic CO_2_ assimilation, which stores the energy of sunlight into reduced carbohydrates—sugars. In the subsequent metabolic reactions, sugars are converted to numerous different biomolecules required by the organism, thus enabling growth (Figure 2).

## 3 Not All the Light Energy Can Be Used by the Cells

Notably, not all the light energy captured by the cells can be effectively used in photosynthesis. Instead, excess energy harvested by the LHC complexes is dissipated from the cells as heat (thermal radiation), which is thereby lost from the system (Demmig‐Adams et al. 2014; Murakami et al. 2024). This protects the photosynthetic cells from light‐induced damage by preventing excited chlorophylls from reacting with oxygen and the production of destructive reactive oxygen species. However, many components involved in this process have been identified (Ruban and Wilson 2021) and engineered for enhanced photosynthetic activity (De Souza et al. 2022; Ghosh et al. 2023); the exact mechanisms behind heat dissipation are still largely unresolved. Plants also protect the photosynthetic machinery from reductive damage by regulating the electron transfer in the ETC through different mechanisms. In photosynthetic control, the Cyt b_6_f complex limits the linear electron flux to PSI and balances the rate of NADPH and ATP production with CO_2_ fixation reactions (Foyer et al. 2012; Degen and Johnson 2024), thereby protecting PSI (Suorsa et al. 2012). In cyclic electron transport (CET) the NDH‐1 complex (Peltier et al. 2016; Laughlin et al. 2020) recycles excess electrons from PSI back to the PQ pool. The electrons can subsequently return to PSI or be removed by PTOX (plastid terminal oxidase), which uses electrons to reduce oxygen and protons back to water (Messant et al. 2024). While the coordination of these complex regulatory circuits, and the roles and interplay of the associated proteins with the key regulator PGR5 (Munekage et al. 2002) have been intensively studied, many aspects are still unclear and under debate (Buchert et al. 2020; Rantala, Lempiäinen, et al. 2020; Degen and Johnson 2024). Whatever the specific mechanisms for energy dissipation are, it is clear that the amount of the overall energy that is lost from the cells (i.e., the energy that is not funnelled into the carbon fixation) depends on multiple combined growth parameters.

## 4 Photosynthesis Is Regulated by the Light Conditions

**FIGURE 3:**
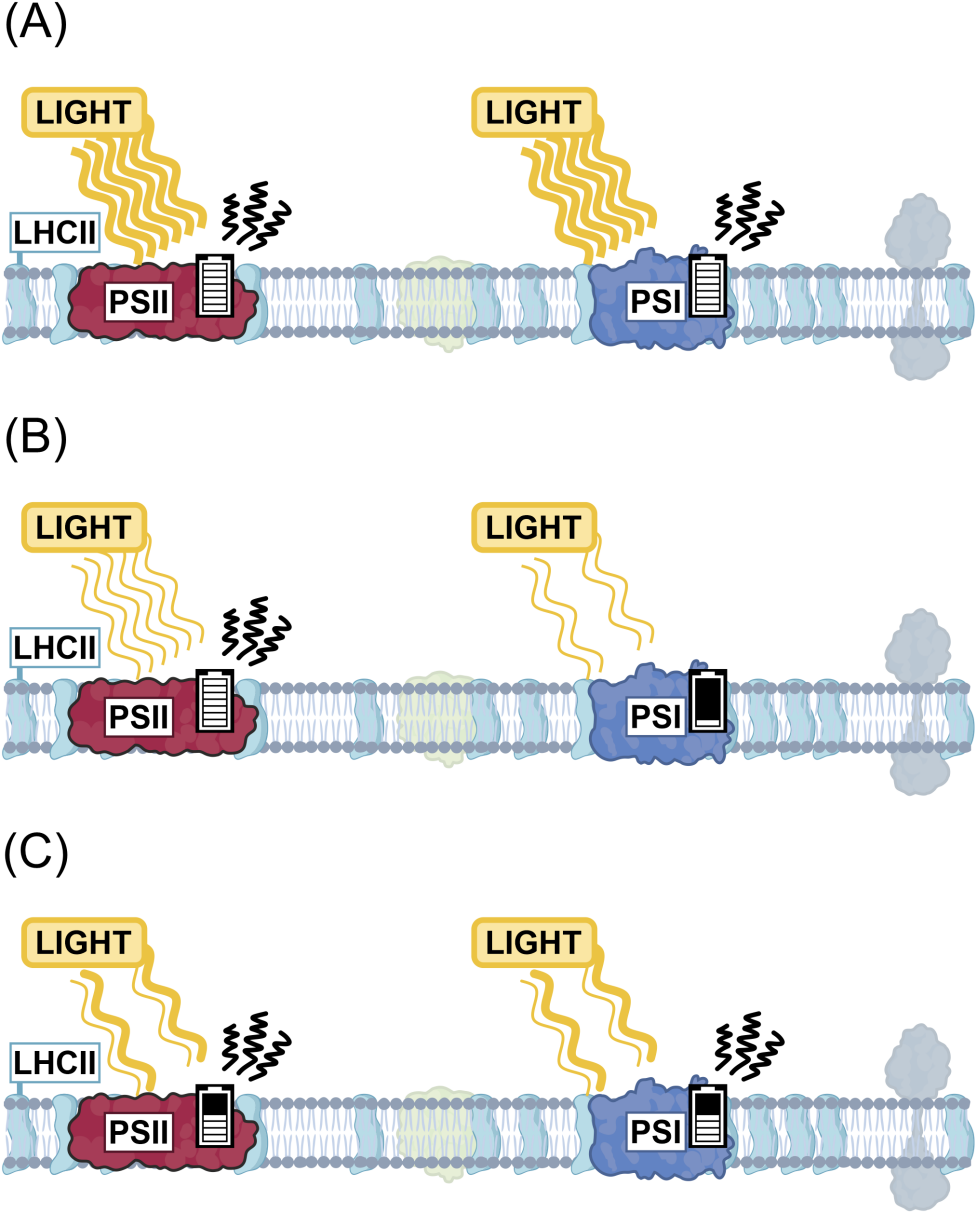
A generalised schematic illustration of suboptimal light scenarios that result in photosynthetic inefficiency and overall energy loss (A) under excessive illumination of PSI and PSII only part of the provided light energy can be used for photosynthesis. (B) Uneven excitation of the photosystems, resulting from different excitation spectra between PSII and PSI, may cause functional imbalance especially under artificial lights. (C) Fluctuating light challenges the function of the photosynthetic machinery and easily leads to PSI damage, that requires energy‐consuming repair and regulatory functions.

Balanced excitation of the two photosystems, PSII and PSI, is the prerequisite for efficient photosynthesis and the integrity of the photosynthetic machinery (Allen et al. 1981; Grieco et al. 2012). Excess light energy may severely damage the photosynthetic machinery (Figure 3A) (Aro et al. 1993) and drain the cell's resources for maintenance and repair, while under limited light, photosynthesis runs below maximum capacity (Figure 3B) thereby reducing productivity (Raven 2011). This is a challenge in nature as the light intensity and spectrum fluctuate according to the diurnal rhythm, cloudiness, reflections in water and the movement of the canopy in the wind (Figure 3C). As plants cannot directly affect the incoming light, they must continuously regulate different molecular processes involved in light harvesting, heat dissipation and distribution between the photosystems.

To adjust the energy uptake, photosynthetic cells reduce the LHC: Photosystem ratio under high light and increase the ratio under low light (Bailey et al. 2001; Wientjes et al. 2013). When a plant acclimates to high‐light conditions, the capacity to dissipate excess energy as heat is increased to protect the cells against photodamage (Tikkanen et al. 2017; Bethmann et al. 2023). However, as a trade‐off, the plant's ability to utilise low‐light intensities is decreased. In the opposite scenario, when a plant acclimates to low‐light conditions, the cells can utilise light more efficiently but are more sensitive to high light‐induced stress. Shifting between these states in response to changing light conditions requires large energy investments from the cell, and collectively, the processes dictate how efficiently the available light energy is used and directed for crop production (Demmig‐Adams et al. 2018). Notably, the light requirements and the ability to acclimate to fluctuations are species‐specific and change during the lifespan of the plant (Ögren and Rosenqvist 1992; Huang et al. 2018; Bethmann et al. 2023).

## 5 Photosynthetic Efficiency Is the Sum of Many Overlapping Factors

Besides the light conditions, there are many overlapping environmental factors that together affect the cell's ability to use captured energy for growth (Sugiura et al. 2019). At the most basic level, the availability of CO_2_ correlates with the capacity of the cell to use light energy productively for the biosynthesis of organic molecules (Stitt 1986). Increased CO_2_ concentration, therefore, boosts growth, but also improves light tolerance and reduces the need for alternative energy‐consuming processes to protect the cell from photodamage (Goh et al. 2012). Higher CO_2_ levels also reduce photorespiration, a futile oxygenation reaction that naturally always competes with the carbon fixation by ribulose‐1,5‐bisphosphate carboxylase/oxygenase (RuBisCo) (Sharkey 1985). At lower temperatures, typically below 25°C, the effect of photorespiration becomes less pronounced, while the efficiency of CO_2_ fixation is enhanced via temperature‐regulated RuBisCo activase (Crafts‐Brandner and Salvucci 2000; Dusenge et al. 2019). The reduction in temperature has also been shown to change the way plants respond to stress caused by high light. For example, in lettuce (*Lactuca sativa*) grown at 23°C, the normal light‐harvesting efficiency is reestablished in seconds after the end of high‐light stress treatment, while at 13°C the recovery takes hours (Lempiäinen et al. 2024). The light spectrum also has an impact on plant CO_2_ uptake, as the phototropin photoreceptors (Phot1 and Phot2) in stomatal guard cells detect blue light (Inoue and Kinoshita 2017) as part of the complex regulation of stomatal opening. Also, this process is co‐regulated by the temperature (Mills et al. 2024) to further control gas exchange and water loss. How changes in light, CO_2_ concentration and temperature affect a plant's ability to perform photosynthesis also depends on the water and nutrient balance (Nam et al. 2021; Asargew et al. 2024), biotic and abiotic stressors (Demmig‐Adams et al. 2013), and the way the plant has experienced all of these factors during its growth. Notably, plants retain a physiological memory of past environmental conditions, which influences their responses to future stressors and resource availability (Liu et al. 2022; Siddique et al. 2024). For example, even transient exposure to chilling temperatures may stunt growth and reduce yield, even if the conditions for the rest of the cultivation period are favourable (Wu et al. 2022).

Under optimal growth conditions, when the productivity is at the maximum, the excitation between the photosystems is in balance, the collected light energy is effectively funnelled to CO_2_ fixation, and the carbon flux to different cellular downstream biosynthetic processes is unobstructed. Because of many simultaneous interlinked variations and complex interactions between the environmental factors and biological response mechanisms, the optimisation of culture conditions for maximising productivity is far from trivial. In order to study the correlations between environmental factors and metabolism, we must be able to alter the different conditions independently in a controlled manner and monitor relevant biological changes in real time.

## 6 Response and Consequences of Using Artificial Light

Because of the natural ability of the photosynthetic machinery to flexibly adjust to prevailing light conditions, it is possible to grow plants under various types of artificial lights, even if the spectral profiles differ significantly from sunlight in their natural habitat. Unnatural light conditions, however, may induce quite dramatic structural changes in the composition of photosynthetic machinery as a consequence of imbalanced excitation of PSII and PSI (Canaani et al. 1982; Leschevin et al. 2024), as well as incorrect signalling from light‐sensing photoreceptors on the plant leaves (Ibrahim et al. 2022; Loudya et al. 2024). This is likely to affect the functional properties and the overall efficiency of the photosynthetic apparatus and is one plausible reason for inconsistencies between experiments conducted in different laboratories. On the other hand, in greenhouses with supplemental lighting, plants have to acclimate between the different spectral properties of artificial and natural sunlight and respond to natural changes in light intensity, which together inevitably reduce the total photosynthetic efficiency.

**FIGURE 4:**
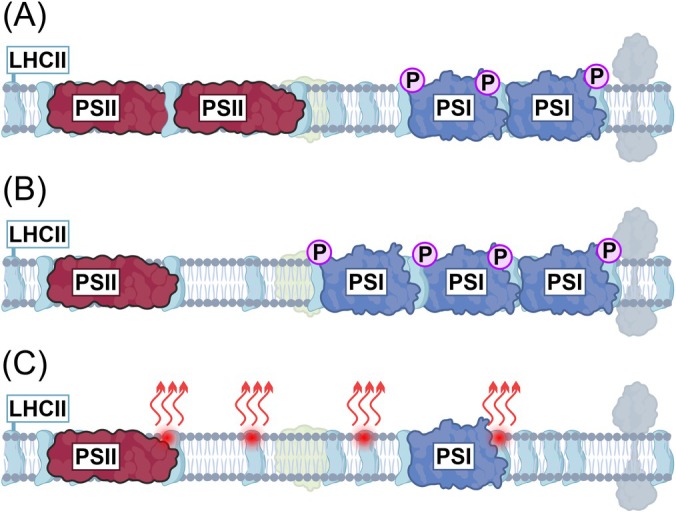
Simplified representation of key photosynthetic response mechanisms involving PSII and PSI that help plants to adjust and compensate for suboptimal light conditions. (A) Phosphorylation of LHCII is a short‐term response to light spectrum overexcitation of PSII relative to PSI that triggers increased LHCII excitation of PSI (B) synthesis of new PSI units as a long‐term response to over excitation of PSII to re‐establish functional balance between the photosystems. (C) Excess excitation energy is dissipated as heat when the plant receives more energy than it can use.

Plants have a variety of molecular mechanisms to balance suboptimal light excitation in order to minimise light‐induced stress and the need for energy‐consuming damage control. Energy distribution to the photosystems is dynamically regulated by specific posttranslational protein modification via phosphorylation and acetylation (Allen et al. 1981; Koskela et al. 2018). When light overexcites PSII relative to PSI, LHCII is highly phosphorylated, which leads to increased energy transfer to PSI (Figure 4A). Long‐term exposure results in changes in the composition of the photosynthetic machinery by increasing the amount of PSI and reducing the amount of PSII (Figure 4B). In reverse, if PSI is overexcited, the cells respond by increasing the amount of PSII (Wagner et al. 2008; Pesaresi et al. 2009). At the same time, the plants adjust the composition of the pigment‐binding proteins in the light‐harvesting antennae, which distribute the energy to the photosystems. The part of the energy that cannot be utilised by the plant under these conditions will be dissipated as heat (Figure 4C) and thus lost. While the overlapping response mechanisms allow plants to grow under a variety of light sources, the overall light use efficiency (LUE) and total photosynthetic yield inevitably remain lower than under light with balanced excitation of PSII and PSI. Collectively, the suboptimal spectrum reduces the energy efficiency of bioproduction and may result in significant crop loss in comparison to lamps designed for balanced excitation of the photosynthetic machinery.

## 7 Complex Dynamic Spectral Effects

**FIGURE 5:**
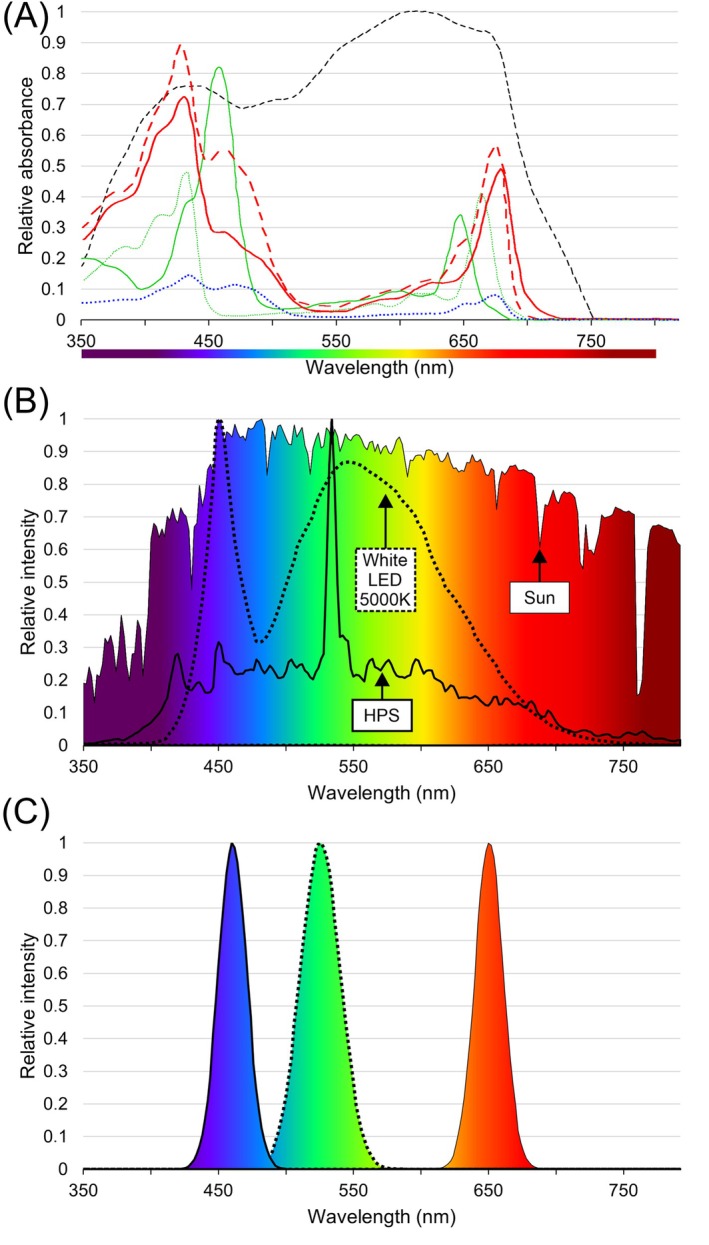
Spectral comparison between different lights and absorption profiles. (A) Illustration of the relative absorbance spectra of plant leaf constituents and photosynthetic pigments. The black dashed line represents the absorbance spectrum of a plant leaf, the green solid and dotted lines correspond to chlorophyll *b* and chlorophyll *a*, respectively (McCree 1981). The red solid and dashed lines represent photosystem I (PSI) and photosystem II (PSII) (redrawn from Wientjes et al. 2013), whereas the blue dotted line represents light‐harvesting complex II (LHCII) (redrawn from Galka et al. 2012). Notably, spectral optimization cannot be based on the spectral properties of the independent molecules, as the absorption and availability of different spectral components is affected by the entire structure of the plant (Figure 1) including the molecular arrangement of the protein complexes in the thylakoid membranes, the composition of the cell layers and the alignment of the leaves. (B) Photosynthetically active region of solar radiation spans the entire visible wavelength range 350–792 nm (thin solid line; colour gradient) while the light from high‐pressure sodium lamps (thick solid line) and white LEDs (dotted line) cover more confined parts of the spectrum. Each spectrum is normalised to its peak emission. (C) The relative spectral intensities of LED lamps consisting of separate red (thin solid line), green (dotted line) and blue (thick solid line) LEDs can be tuned to balance the excitation between PSII and PSI for improved photosynthetic performance.

PSII, PSI and different LHC complexes all have different absorption spectra (Figure 5A) (Brown and Schoch 1981), which have a direct impact on the way the photosynthetic machinery responds to light. In the generalised traditional view, blue light (~400–450 nm) and red light (~620–680 nm) excite PSII, whereas far red light (~700–800 nm) excites PSI (Figure 5A). It is tempting to assume that there is some desired specific ratio between these wavelengths that would provide maximum photosynthetic efficiency and optimal productivity. In reality, the interactions are highly complex and dynamic, and even small changes in the wavelength profiles and intensity can have a significant impact on how the light reactions are excited in proportion to one another (Mattila et al. 2020). When the light penetrates the canopy, the radiation reaching the leaves at the top is very different from the light reaching leaves at the bottom, and the wavelength profile is no longer uniform or as clearly defined (Sellaro et al. 2025). Thus, the proportions of the available wavelengths change in the different layers and tissue types of the plant, which is further complicated by the overlap between the different spectral effects. While red and blue wavelengths are effectively absorbed and used in the top layers of the canopy, green light, which is generally used less effectively, penetrates deep through the vegetation with a significant role in promoting total photosynthesis (Lanoue et al. 2022). Because of these effects, the optimum spectrum depends not only on the photosystem properties, but also on the plant structure, age and tissue type. Consequently, the light spectrum is always setup‐dependent, and there is no single generic wavelength profile that would be ‘optimal’ for plant growth (Figure 5) (Zhen and Bugbee 2020).

## 8 Light Fluctuation

In nature, light is rarely constant but may fluctuate significantly depending on the movement of clouds, shading leaves or reflections in water, thus potentially spanning a wide range of rapidly changing light intensities and quality (Sellaro et al. 2025). This is a demanding regulatory challenge to plants and requires constant adjustment of the photosynthetic machinery to maintain balanced photosystem excitation and minimise the damaging effect of excess light (Figure 3C). Mutant plants that are unable to adjust the distribution of excitation energy between PSII and PSI do not necessarily have a visual phenotype under constant light but may be non‐viable under rapidly changing light intensities (Tikkanen et al. 2010). Such even lethal phenotypes have been associated specifically with damage to PSI (Grieco et al. 2012; Suorsa et al. 2012), and this has spurred research on the mechanisms by which different lineages of photosynthetic organisms protect PSI from fluctuating light (Allahverdiyeva et al. 2015). Importantly, if the spectrum of fluctuating light differs too much from natural light, the balancing capacity of the photosynthetic machinery may be exceeded, which may lead to the destruction of PSI in WT plants (Tikkanen and Grebe 2018).

Although in nature the lower leaves are more exposed to fluctuating light due to plant movements in the wind, the light filtered through the leaves is dominated by far‐red wavelengths (Sellaro et al. 2025). PSI naturally has a higher capacity to use such far‐red canopy light than PSII (Figure 5A), which promotes ETC oxidation under shading leaves and alleviates the risk of overreduction and PSI damage upon high‐intensity flashes (Kono et al. 2017) (Figure 4A). Simultaneously, LHCII phosphorylation ensures that PSI receives sufficient light energy under neutral shadow light, further protecting PSI from reductive damage during light fluctuations (Grieco et al. 2012) (Figure 4B). Problems occur when PSII is overexcited in comparison to PSI (Figure 3B), which results in ETC overreduction and exposes PSI to severe damage upon high‐light flashes (Tikkanen and Grebe 2018). This is because the mechanisms that would normally protect PSI under high light by controlling electron transfer through Cyt b_6_f are not active under the prevailing low light and do not have time to activate during short light pulses. These effects are being studied to understand the native regulatory interactions in plants, but as the light spectrum of the artificial lights differs from natural light, the conclusions are not necessarily physiologically relevant.

## 9 Artificial Light Confuses Natural Chloroplast Signalling in Plants

The chloroplast that contains the photosynthetic machinery is at the center of the plant's energy metabolism and interacts with the nucleus and other parts of the plant cell via complex signalling cascades (Kangasjärvi et al. 2014; Gollan et al. 2015; Chan et al. 2016). Chloroplast signalling ensures that required nucleus‐encoded transcripts are produced and transported to the chloroplast, but also affects how the cell responds to environmental changes and different types of stress conditions. This signalling is largely regulated based on the redox state of the photosynthetic machinery, influenced by the electron transport rate (ETR) between PSII and PSI, and the capacity of the stroma to accept electrons (Gollan et al. 2017). Although natural white light has a balanced effect on the ETR and stromal redox state, artificial light may easily disturb the native regulation. For example, LED lamps used for growing plants are typically enriched in red light that overexcites PSII and results in PQ reduction (Figure 5B,C) (similar to what is observed in high‐light conditions), while stroma remains oxidised (as in low‐light conditions) (Piippo et al. 2006). Such effects easily lead to unnecessary stress responses, reduced capacity to utilise light for photosynthesis (Demmig‐Adams et al. 2013), and ultimately decreased crop yield. Notably, besides the unwanted effects, manipulation of the chloroplast signalling by light can be used for modulating the synthesis of specialised metabolites that may have nutritional, taste‐related or pharmacological value, or protect the plant against biotic stresses threatening the crop (Fernandez and Burch‐Smith 2019; Tóth et al. 2024). These responses overlap and are dependent on the species and the developmental stage of the plant.

## 10 Artificial Light and LED Technologies

The development of LED technology in the recent decade has provided access to more economical, energy‐efficient light systems for greenhouses and indoor farming than traditional metal halide or high‐pressure sodium (HPS) lamps (Sena et al. 2024). LED lights are relatively cheap to manufacture, have higher electricity‐to‐light conversion efficiency, require low maintenance, and can have adjustable intensity and colour range. However, the LEDs that are available on the market have been primarily designed based on the characteristics and light perception of the human eye, not photosynthesis. Consequently, the typical LED light spectrum does not mimic natural light, but instead is composed of several narrow emission peaks that may be far from optimal photosynthetic radiation (Figure 5B,C). As a result, LEDs do not excite the photosystems in a balanced manner as sunlight does, potentially leading to suboptimal photosynthetic performance. In LEDs, the type and composition of the LED semiconductor material determine the light wavelength, which can be manufactured to range from ultraviolet (~260 nm) to infrared (~950 nm). Most typical single LED elements are either red, blue or green (Figure 5C), whereas the white elements are obtained by phosphor‐coating to shift the output wavelength towards yellow (Figure 5B). The colour spectrum of a multicomponent lamp can be adjusted based on the combined LED emission properties, and could be used to generate lights that would be more optimal for the photosynthetic machinery. Although the natural white light spectrum cannot be replicated with LEDs, balanced excitation of the photosystems could be achieved by specific combinations of LED emission wavelengths. This, however, still requires in‐depth collaborative research and technology development to compare the molecular and physiological responses of plants under artificial and natural light.

In commercial indoor farming, the balanced PSII‐PSI excitation as such has no value, but the most important factor is the yield efficiency (YE), which describes how efficiently the input electricity supports plant growth and leads to a commercially valuable harvest. YE is the sum of the efficiency of the electricity‐to‐light conversion (electricity efficiency; EE), which is dictated by the lamp, and the efficiency at which the plant's photosynthetic machinery converts the light energy into chemical energy LUE. Importantly, although LUE is usually far from optimal, sufficient YE is achieved by high EE and the lamp's low cost (i.e., price relative to lifespan). The electricity used for artificial lighting is a major operational cost in indoor farming, and, based on the current understanding of the photosynthetic machinery, there is room to improve photosynthetic output while minimising energy consumption. From the viewpoint of LUE, there is currently significant overall energy loss resulting from (i) excess or wrong type of light that cannot be used by plants, (ii) inefficient photosynthesis operating below maximum due to unbalanced excitation of the photosystems and (iii) cellular resources that are wasted in the protective molecular mechanisms and repair under induced light stress.

## 11 Monitoring the Process: Analysing Photosynthesis

In order to evaluate the plant growth efficiency and the overall culture system performance, we must be able to measure different photosynthetic functions in intact plants. This is typically done using two alternative methods. Monitoring CO_2_ uptake from the air gives a direct indication of the net photosynthetic rate of the plant, which ultimately correlates with growth. CO_2_ uptake is typically measured using small chambers or leaf clamps from a selected part of the plant, and it provides information on the rate of photosynthesis, but nothing about the factors that affect the rate under different conditions. The other method is based on measuring chlorophyll fluorescence—light emission that corresponds to excess energy dissipated by the chlorophyll molecules alongside thermal radiation. Chlorophyll fluorescence gives information on the mechanisms that affect the photosynthetic efficiency, excitation energy distribution, and electron transfer around PSII (Baker 2008; Tikkanen et al. 2017). We can measure passive fluorescence induced by the ambient light, but this does not provide any specific useful information on the efficiency of photosynthesis. There are various fluorescence‐based analytical methods used in plant research, which provide detailed functional data on photosynthesis from intact plants (Maxwell and Johnson 2000; Kolber et al. 2005; Küpper et al. 2019; Porcar‐Castell et al. 2021). The most commonly used method is pulse‐amplitude modulated (PAM) fluorometry that is used to measure four basic parameters: fluorescence in near darkness (F0), fluorescence during a saturating light pulse in darkness (Fm), light‐induced fluorescence (F′) and light‐induced fluorescence (Fm′) (for further reading, see Murchie and Lawson 2013). This information could be used for condition optimisation to improve photosynthetic productivity, but there are technical challenges in performing the measurements in the production environment in real time, as PAM requires adaptation to darkness, specific wavelengths and very high‐intensity light pulses.

## 12 Technology Development Towards Next‐Generation Adjustable Cultivation Platforms

To study the photosynthetic performance and plant behaviour in basic research, and to improve the energy efficiency of indoor farming, we need systems that allow us to (i) flexibly alter different culture parameters, (ii) monitor the response at the plant and molecular level and (iii) respond to these changes in real time. To achieve this, we must have precise control over the plant growth conditions, including light quality and intensity, CO_2_ concentration, humidity, temperature and irrigation. For research purposes, the instrumentation must enable a very broad range of light wavelengths and intensities for the study of photosynthetic responses that will also include a variety of unnatural and extreme conditions. This makes it possible to assess the complex native response mechanisms and the connections between the molecular photosynthetic functions and the overall plant behaviour at the macro level, which is needed for future rational crop engineering and more precise growth optimisation. The available commercial culture chambers typically allow the control of basic growth parameters, but do not necessarily have integrated irrigation systems or ways to collect data on photosynthesis by monitoring CO_2_ consumption or chlorophyll fluorescence. At the commercial greenhouse scale, the development of LED technologies could provide benefits through more careful selection and fine tuning of the light spectra and intensity based on the crop and the specific instrumentation. Currently, we have extensive biological and technical expertise to take these fields forward, but it requires close collaboration and fluent communication between academia and industry, the plant researchers, LED manufacturers and farmers who utilise the systems. Measuring and interpreting the biological data from the plants is an interlinked challenge with significant development potential. More accurate real‐time monitoring systems that continually provide information on photosynthetic efficiency, productivity, possible constraints and the well‐being of the plant can be used to develop automated optimisation systems. Integrated measuring systems—including multispectral and thermal camera technology (Pineda et al. 2021; Xia et al. 2023) and fluorescence imaging (Gorbe and Calatayud 2012)—are now being established for advanced data acquisition (Gill et al. 2022; Walsh et al. 2024). Combined with AI‐based advanced data processing systems, it would provide tools for multiparameter feedback regulation to maximise crop yield with minimal energy input. Besides the biologists and engineers, this interdisciplinary mission is dependent on the combined effort of machine learning specialists, modellers and software developers to convert the acquired data into response systems applicable in laboratory research and ultimately in large‐scale indoor farming.

## Author Contributions

M.T. initiated the manuscript and wrote the first draft. P.K. then developed this draft into the current form of the article. G.K. and S.P. were responsible for the illustrations, including their captions. All authors contributed to the refinement and finalization of the manuscript.
